# In Vitro Replication Inhibitory Activity of Xanthorrhizol against Severe Acute Respiratory Syndrome Coronavirus 2

**DOI:** 10.3390/biomedicines9111725

**Published:** 2021-11-19

**Authors:** Minwoo Kim, Hee Cho, Dae-Gyun Ahn, Hae-Gwang Jung, Han Young Seo, Ji-Su Kim, Youn-Jung Lee, Jun Yong Choi, In Ho Park, Jeon-Soo Shin, Seong-Jun Kim, Jong-Won Oh

**Affiliations:** 1 Department of Biotechnology, Yonsei University, 50 Yonsei-ro, Seodaemun-gu, Seoul 03722, Korea; godspeed88@hanmail.net (M.K.); heecho@yonsei.ac.kr (H.C.); dgahn@krict.re.kr (D.-G.A.); primary21@naver.com (H.-G.J.); cuhoo123@yonsei.ac.kr (H.Y.S.); smile7530@naver.com (J.-S.K.); 2 Division of Infectious Diseases, Department of Internal Medicine, Yonsei University College of Medicine, Seoul 03722, Korea; YJLEE00@yuhs.ac (Y.-J.L.); SERAN@yuhs.ac (J.Y.C.); 3 Severance Biomedical Science Institute, Yonsei University College of Medicine, Seoul 03722, Korea; INHOPARK@yuhs.ac (I.H.P.); JSSHIN6203@yuhs.ac (J.-S.S.); 4 Department of Microbiology, Yonsei University College of Medicine, Seoul 03722, Korea; 5 Center for Convergent Research of Emerging Virus Infection, Korea Research Institute of Chemical Technology, Daejeon 34114, Korea; sekim@krict.re.kr

**Keywords:** SARS-CoV-2, xanthorrhizol, pan-coronavirus antivirals, herbal medicine, SARS-CoV-1, human coronavirus

## Abstract

In spite of the large number of repositioned drugs and direct-acting antivirals in clinical trials for the management of the ongoing COVID-19 pandemic, there are few cost-effective therapeutic options for severe acute respiratory syndrome (SARS) coronavirus 2 (SCoV2) infection. In this paper, we show that xanthorrhizol (XNT), a bisabolane-type sesquiterpenoid compound isolated from the *Curcuma xanthorrhizza* Roxb., a ginger-line plant of the family *Zingiberaceae*, displays a potent antiviral efficacy in vitro against SCoV2 and other related coronaviruses, including SARS-CoV-1 (SCoV1) and a common cold-causing human coronavirus. XNT reduced infectious SCoV2 titer by ~3-log_10_ at 20 μM and interfered with the replication of the SCoV1 subgenomic replicon, while it had no significant antiviral effects against hepatitis C virus and noroviruses. Further, XNT exerted similar antiviral functions against SCoV2 variants, such as a GH clade strain and a delta strain currently predominant worldwide. Neither SCoV2 entry into cells nor the enzymatic activity of viral RNA polymerase (Nsp12), RNA helicase (Nsp13), or the 3CL main protease (Nsp5) was inhibited by XNT. While its CoV replication inhibitory mechanism remains elusive, our results demonstrate that the traditional folk medicine XNT could be a promising antiviral candidate that inhibits a broad range of SCoV2 variants of concern and other related CoVs.

## 1. Introduction

Following the outbreak of severe acute respiratory syndrome (SARS) in November 2002, a second SARS coronavirus (CoV), SCoV2 emerged in December 2019 in the Hubei province in China [1]. SCoV2 is believed to have originated from bat-origin SARS-related coronavirus and introduced into the human network via an intermediate host, such as pangolins [2]. Despite the social distancing, massive diagnosis, and self- or forced quarantine, the World Health Organization (WHO), concerning the rapid spread of this zoonotic virus at an unprecedented speed, declared a pandemic of SCoV2 in April 2020. The SCoV2 spillover into human networks has been imposing a huge burden on the healthcare sector because the risk of mortality in patients infected with SCoV2 is much higher (~2%) than that of circulating, conventional CoVs or influenza virus [3,4].

SCoV2, which belongs to the family of *Coronaviridae*, has a positive-sense RNA genome of ~31 kb [5]. The RNA genome is replicated by the replicase polyproteins (pp) encoded by ORF1a and ORF1b. The nonstructural protein (nsp) pp1ab, which is produced by −1 programmed ribosomal frameshifting, is processed by the nonstructural protein 3 (Nsp3) with papain-like protease (PLpro) activity and the 3C-like (3CL) protease Nsp5 to produce a total of 16 viral nsps. As in other CoVs, SCoV2 replication produces a set of subgenomic (sg) mRNAs to express viral structural proteins (spike (S), envelope (E), membrane (M), and nucleocapsid (N) proteins) and the accessory proteins, some of which might be involved in the viral invasion of host innate antiviral immune responses [6]. During the epidemics of SCoV1 in 2003, various antiviral agents, including interferons (IFNs), ribavirin, and lopinavir/ritonavir, were used for the treatment of SARS [7,8]. However, no effective or specific antiviral therapies have yet been developed and officially approved for uses in a clinical setting against SCoV1 and the newly emerged SCoV2, except remdesivir (RDV), a viral RNA polymerase inhibitor currently approved by the US Food and Drug Administration (FDA) [9].

Herbal medicines have been used for thousands of years to treat diverse diseases, including microbial infections, metabolic diseases, and cancers [10]. Moreover, recent studies have highlighted the potential use of traditional herbal medicine for the treatment of coronavirus disease 2019 (COVID-19) [11,12,13]. *Curcuma xanthorrhizza,* which is cultivated in Indonesia on an industrial scale and in some other Southeast Asian countries (Malaysia, Thailand, the Philippines, and Vietnam), and its extracts have been widely used for the treatment of diverse diseases in Indonesia, India, and China [14]. Xanthorrhizol (XNT; C_15_H_22_O, 2-methyl-5-[(2R)-6-methylhept-5-en-2-yl]phenol), also known as Java turmeric, is a major constituent of the rhizomes of *Curcuma xanthorrhizza* Roxb., a ginger-like plant of the family *Zingiberaceae* [15]. XNT can be either extracted from the *Curcuma* rhizome or chemically synthesized using simple starting materials [14,16]. It is known to possess a variety of biological activities, such as anticancer, antimicrobial, anti-inflammatory, antioxidant, antihyperglycemic, antihypertensive, antiplatelet, nephroprotective, hepatoprotective, and estrogenic and/or anti-estrogenic effects [15,17]. Curcumin, another ingredient abundantly present in *Curcuma* extracts, and its derivatives exhibited promising antiviral activity against dengue virus (DENV), Zika virus (ZIKV), chikungunya virus, hepatitis C virus (HCV), and SCoV2 [18,19,20,21]. A previous study showed that crude *Curcuma* extracts also inhibit HCV entry and possibly viral replication [22]. However, little is known about the antiviral potential of a single compound XNT isolated from *Curcuma* extracts. Moreover, its antiviral activity has not yet been tested against SCoV2 as well as other CoVs.

Currently, there are few cost-effective therapeutic options for the treatment of COVID-19. Considering the high cost of approved drugs, including RDV, for those in regions where many people cannot access effective prophylactic or therapeutic antiviral drugs for the treatment of patients with severe symptoms of COVID-19, alternative medicines are urgently needed. In this study, we aim to evaluate XNT as an antiviral agent against SCoV2. We show that XNT significantly reduces the viral loads of SCoV2. XNT also inhibited SCoV1 and a common cold-causing human coronavirus 229E (HCoV-229E), demonstrating its broad-spectrum antiviral activity against CoVs.

## 2. Materials and Methods

### 2.1. Reagents, Plasmids, and Antibodies

XNT (≥97% purity) and ebselen were purchased from Cayman Chemical (Ann Arbor, MI, USA). XNT was dissolved in 100% DMSO and added to the culture media or each enzymatic reaction or SCoV2 Spike-pseudotyped virus cellular entry assay at a final concentration of 0.1% or 1–4%, as specified in figure legends. RDV was obtained from MedChemExpress (MCE; Monmouth Junction, NJ, USA). Bismuth citrate and E-64d (cysteine protease inhibitor, E8640) were purchased from Sigma-Aldrich (Saint Louis, MO, USA). Hydroxychloroquine (HCQ, S4430) was obtained from Selleckchem (Houston, TX, USA).

pSARS-REP-Feo plasmid, a SCoV1 subgenomic replicon that expresses firefly luciferase (Fluc) reporter, was described previously [23]. Antibodies were obtained as follows: mouse monoclonal anti-α-tubulin antibody (clone DM1A) from Calbiochem (La Jolla, CA, USA); rabbit polyclonal anti-β-actin antibody (#4967) from Cell Signaling (Beverly, MA, USA); mouse monoclonal anti-SCoV2 nucleocapsid (N) antibody (40143-MM08) from SinoBiological (Beijing, China); and mouse monoclonal anti-SCoV1/SCoV2 anti-S antibody (clone 1A9) from GeneTex (Irvine, CA, USA).

### 2.2. Cell Culture

African green monkey kidney cell line Vero E6, human lung adenocarcinoma cell line Calu-3, human embryonic kidney 293 (HEK293) and human embryonic kidney-derived HEK293T cells, human fetal lung fibroblast MRC-5, and murine macrophage cell line RAW264.7 were cultivated in Dulbecco’s modified Eagle’s medium (DMEM) supplemented with 10% fetal bovine serum (FBS), 100 U/mL of penicillin, and 100 μg/mL streptomycin. Human hepatocellular carcinoma cell line Huh7 was grown in DMEM supplemented with 10% FBS, 2 mM L-glutamine, 100 U/mL of penicillin, 100 μg/mL of streptomycin, and 0.1 mM of nonessential amino acids. The Huh7-derived cell lines R-1 and HG23, which harbor a self-replicating HCV subgenomic replicon [24] and a Norwalk virus subgenomic replicon [25], respectively, were grown in the same medium supplemented with G418. All cultures were maintained under standard culture conditions (5% CO_2_, 37 °C).

### 2.3. Viruses and Plaque Assay

SCoV2 strain KCDC03 (SARS-CoV-2/human/KOR/KCDC03/2020; GenBank accession number MT020782 and GISAID accession number EPI_ISL_407193, accessed on 11 March 2020) was obtained from the National Culture Collection for Pathogens (NCCP), South Korea. The GH clade SCoV2 YS006 (SARS-CoV-2/human/KOR/YS006/2020; GenBank accession number MW345824 and GISAID accession numbers EPI_ISL_660109, accessed on 8 December 2020) isolated from nasopharyngeal swabs from patients with COVID-19 in South Korea was reported previously [26]. A SCoV2 delta strain YS117 (GenBank accession number MZ798798 and GISAID accession number EPI_ISL_3411836, accessed on 15 August 2020) was isolated from a clinical sample as described previously [26]. The study was approved by the institutional review board (IRB) of Severance Hospital, Yonsei University Healthcare System, with written informed consent from the patients (IRB protocol number 4-2020-0076, 21 March 2020). These CoV stocks were propagated in Vero E6 cells grown in DMEM supplemented with 2% FBS. Infectious virus titer was determined by plaque assay as described previously [27]. Briefly, Vero cells seeded in a 6-well plate were inoculated with 10-fold serially diluted virus samples in a serum-free medium by incubation for 1 h. After washing with PBS, cells were overlaid by SeaPlaque agarose (1% w/v; Lonza, Rockland, ME, USA) in DMEM supplemented with 2% FBS, 100 U/mL of penicillin, and 100 μg/mL streptomycin. After 3–4 days, when visible plaques are formed, cells were fixed with 10% formaldehyde and stained with 1% crystal violet. All the experiments using live SCoV2 were conducted in a biosafety level 3 (BL3) facility with powered air-purifying respirators at the Avison Biomedical Research Center (ABMRC, Yonsei University College of Medicine; Institutional Biosafety Committee (IBC) permit numbers A-202009-260-01, 22 September 2020). Other works using SCoV2 genes were approved by the IBC at Yonsei University College of Medicine (IBC-2020-008, 11 May 2020).

Human coronavirus 229E (HCoV-229E; ATCC VR-740) was propagated in MRC-5 cells (IBC-A-202108-285-01; approved by the IBC of Yonsei University, 6 August 2021). Plaque assay was performed as described previously [28] with slight modifications. Briefly, the 10-fold serially diluted virus samples were inoculated onto Huh7 cells. After 1 h incubation, the cells were overlaid with 0.6% SeaPlaque agarose in DMEM supplemented with 2% FBS, 100 U/mL of penicillin, and 100 μg/mL streptomycin. Murine norovirus (MNV-1.CW1 strain, a gift from Herbert W. Virgin (Washington University School of Medicine, St. Louis, MO, USA)) was propagated in RAW264.7 cells. Viral titer was determined by plaque assay as previously described [29].

### 2.4. Cell Viability Assay

Cytotoxicity of XNT and other antiviral compounds were measured using MTS (3-(4,5-dimethylthiazol-2-yl)-5-(3-carboxymethoxyphenyl)-2-(4-sulfophenly)-2H-tetraxolium) reagent (Promega, Madison, WI, USA). Briefly, cells grown in a 96-well plate (2 × 10^4^ cells/well) were treated with various concentrations of compounds for 24 or 48 h. After 1 h incubation at 37 °C following addition of MTS, the absorbance was read on a 96-well microplate reader (GloMax-Multi Detection System, Promega).

### 2.5. Real-Time Reverse-Transcription Quantitative PCR (RT-qPCR)

The total RNA was isolated using Trizol reagent (Invitrogen, Carlsbad, CA, USA). Copy numbers of HCoV-229E genomic RNA (gRNA), SCoV1 N-coding subgenomic (sg) mRNA, and HCV subgenomic RNA were determined using Realtime PCR Master Mix (Toyobo, Osaka, Japan), as described previously [23,24,30]. The MNV-1 gRNA and Norwalk virus replicon RNA (HG23) levels were determined using TOPreal qPCR 2X PreMIX (Enzynomics, Daejeon, South Korea) [29]. The primers used for RT-qPCR are listed in Supplementary Table S1. Standard RNAs for SCoV2 gRNA and SCoV1 sgRNA were prepared by in vitro transcription using T7 MEGAscript kit (Ambion, TX, USA) and PCR-amplified cDNA templates. For the determination of the SCoV2 gRNA copy number, a template representing a specific region of ORF1ab was RT-PCR-amplified using a forward primer (5′-TAATACGACTCACTATAGATCATCCAAATCCTAAAGGATTTTG-3′; the sequence underlined is T7 promoter) and a reverse primer (5′-CGACATCAGTACTAGTGCCTGT-3′) [31]. RT-qPCR quantification of SCoV1 replicon RNA and SCoV1 replicon-derived N-specific sgRNA was described previously [23].

### 2.6. SCoV2 Entry Assay

The murine leukemia virus (MLV)-based SCoV2 spike protein (S)-pseudotyped retrovirus (SARS2pp) was used for SCoV2 entry assay. The pseudovirus was generated, as described previously [32] with some modifications, using the plasmids as follows: pUMVC (Addgene plasmid #8449; Addgene, Watertown, MA, USA), a packaging plasmid including the MLV *gag-pol*; pBABE-puro-NanoLuc, a retroviral expression vector generated by inserting the Nano luciferase-coding gene (PCR-amplified from the pNL1.1.TK[Nluc/TK] (Promega)) into the retroviral transfer plasmid pBABE-puro (Addgene, plasmid #1764) using In-Fusion HD Cloning Kit (Takara, Kyoto, Japan) following linearization of the vector with HindIII; pcDNA3.1_SCoV2-SΔC19, a mammalian vector with a human-codon-optimized cDNA encoding SCoV2 S protein with a deletion of the C-terminal 19-amino acids ER-retention signal [33]. The mixture of three plasmids was introduced into HEK293T cells by calcium phosphate-mediated transfection. After media change 12 h post-transfection, culture media containing pseudovirus were harvested 2 days later, centrifuged, and passed through a 0.22 µm syringe filter. The resulting SARS2pp was transduced into HEK293T cells transiently expressing the SCoV2 entry receptor human ACE2 (hACE2) [34]. After media change 12 h later, the cells were incubated for 48 h and then lysed in a Glolysis buffer (Promega) for a luciferase assay using the Nano-Glo luciferase assay system (Promega).

### 2.7. SCoV1 Subgenomic Replicon Replication Assay

pSARS-REP-Feo and pRL-TK (Promega) expressing *Renilla* luciferase (Rluc) (used as an internal control) were introduced into HEK293 or HEK293T cells by calcium phosphate-mediated transfection. After 6 h, the cells were washed, treated with indicated concentrations of XNT or DMSO vehicle only, and further incubated for 24 h. Rluc and Fluc activities were measured using the Dual-Glo luciferase assay system (Promega).

### 2.8. Immunoblotting Analysis

Cells were lysed in a lysis buffer (50 mM Tris–HCl, pH 7.5, 150 mM NaCl, 1 mM EDTA, and 1% Triton X-100) supplemented with an EDTA-free protease inhibitor cocktail (Roche Diagnostics, Mannheim, Germany) by incubating on ice for 20 min. The cleared cell lysates were resolved by sodium dodecyl sulfate-polyacrylamide gel electrophoresis (SDS-PAGE), transferred to the nitrocellulose Hybond ECL membrane (GE Healthcare Life Sciences, Piscataway, NJ, USA), and subjected to immunoblot analysis using appropriated antibody sets.

### 2.9. Expression and Purification of Recombinant SCoV2 Enzymes

The *E. coli* codon-optimized cDNAs for the SCoV2 (Wuhan strain, NC_045512.2) Nsp12, Nsp5, and Nsp13 were chemically synthesized, PCR-amplified using specific primer sets, and cloned into an expression vector pTrcHisB (Invitrogen). To prepare Nsp5 with its N-terminal end, a factor-Xa cleavage site was inserted between the (His)_6_-tag and Nsp5. Expression and purification of the recombinant SCoV2 enzymes were carried out as described previously [35]. Briefly, all of these enzymes were expressed in *E. coli* Rosetta strain (Sigma-Aldrich). The cells transformed with each expression vector were grown at 37 °C, and protein expression was induced for 20 h at 16 °C by adding 0.5 mM isopropyl-β-D-thiogalactopyranoside (IPTG). The cells were collected, resuspended in a binding buffer, and sonicated to collect the aqueous phase, which was subjected to metal affinity chromatography using a Ni-nitrilotriacetic acid (Ni-NTA) agarose resin (Qiagen, Hilden, Germany). The protein-containing fractions were collected and dialyzed against buffer A (50 mM Tris–HCl, pH 8.0, 50 mM NaCl, 1 mM DTT, and 10% glycerol). If needed, the proteins were further purified by using a Q-Sepharose column (Amersham Biosciences, Piscataway, NJ, USA). For Nsp5, the purified protein was digested with factor Xa (New England Biolabs, Ipswich, MA, USA) overnight at 20 °C in buffer A and the mixture was reloaded onto Ni-NTA agarose resin to collect the flow-through fraction containing Nsp5 protein. All the purified proteins were dialyzed against buffer A before storage of aliquots at −80 °C until used in enzyme assays.

### 2.10. Nsp12 RdRp Assay

The in vitro RNA-dependent RNA polymerase (RdRp) assay was performed as described previously [35]. Briefly, a reaction mixture containing 3 pmol of purified recombinant SCoV2 Nsp12, 1 μg of poly(C) as a template, 10 pmol of oligo(G)_20_ as a primer, 5 μM GTP, and 5 μCi [α-^32^P]-GTP (Amersham Pharmacia Biotech, Uppsala, Sweden) in a total volume of 25 μL RdRp reaction buffer (50 mM Tris–HCl, pH 8.0, 50 mM NaCl, 2 mM MnCl_2_, 1 mM DTT, 10% glycerol, and 20 U of RNase inhibitor) was incubated for 1–2 h at 32 °C. The reaction products of the RdRp assays were resolved on a denaturing 7.5% polyacrylamide gel containing 8 M urea. After electrophoresis, the gel was exposed to a phosphorimaging plate, and the reaction products were visualized using an Amersham Typhoon 5 Biomolecular Imager (GE Healthcare Life Sciences).

### 2.11. Nsp5 Protease Assay

A fluorescence resonance energy transfer (FRET)-based protease assay was performed as described previously [36]. The enzyme reaction was performed in a black-and-flat-bottomed 96-well microtiter plate with 100 nM purified recombinant SCoV2 Nsp5 in a 100 μL reaction buffer (50 mM Tris buffer, pH 7.5). After preincubation for 10 min at room temperature with an inhibitor at different concentrations, the FRET substrate peptide, DABCYL-KTSAVLQ↓SGFRKME-EDANS, was added to a final concentration of 10 μM and the reaction mixture was further incubated for the indicated time periods at 25 °C. The readouts from the same reactions without Nsp5 were measured as blanks. The fluorescence intensities emitted from EDANS after excitation at 340 nm were measured at 535 nm in a fluorometer (Victor 5; PerkinElmer Biosciences, Boston, MA, USA).

### 2.12. Nsp13 Helicase Assay

A FRET-based helicase unwinding assay by Nsp13 was performed as previously described [37] with slight modifications. The DNA unwinding assay was performed by incubating 80 nM of purified SCoV2 Nsp13, 100 nM of DNA duplex substrate prepared by annealing FL-BHQ oligo (5′-TTTTTTTTTTTTTTTTTTTTCGAGCACCGCTGCGGCTGCACC-BHQ1-3′) and RL-FAM oligo (5′-FAM-GGTGCAGCCGCAGCGGTGCTCG-3′) (Macrogen, Seoul, South Korea), and 125 nM capture strand (5′-GGTGCAGCCGCAGCGGTGCTCG-3′) in a 100 μL reaction buffer (50 mM Tris–HCl, pH 7.5, 0.075% Triton X-100, and 1 mM ATP), in a 96-well black polystyrene plate. The reactions were incubated for 60 min at 25 °C, and the fluorescence signals excited at 485 nm wavelength were measured at 535 nm using a fluorometer (Victor 5; PerkinElmer Biosciences).

### 2.13. Interferon β Reporter Assay

HEK293T or Vero E6 cells grown in 24-well plates were co-transfected with pGL3-IFN-β (500 ng) (provided by John Hiscott (McGill University, Montreal, QC, Canada)) [34] and pRL-TK (50 ng). After 6 h, fresh media containing XNT or DMSO vehicle (0.1% final concentration) were added to the transfected cells prior to measuring Fluc and Rluc activities 24 h later, using the Dual-Glo luciferase assay system (Promega).

### 2.14. Data Analysis and Statistical Analysis

The 50% effective concentration (EC_50_) values for the inhibition of CoV replication by antiviral compounds were determined using GraphPad Prism 6.01 (GraphPad Prism Software Inc., La Jolla, CA, USA). Data in this study are presented as mean ± standard deviation (SD) from at least three independent experiments, unless otherwise stated. Statistical analysis was performed using GraphPad Prism 6.01. The *p*-value was calculated using the unpaired Student′s *t*-test, and those with *p* < 0.05 were considered statistically significant.

## 3. Results

### 3.1. Identification of XNT as an Antiviral Agent against SCoV2

**Figure 1:**
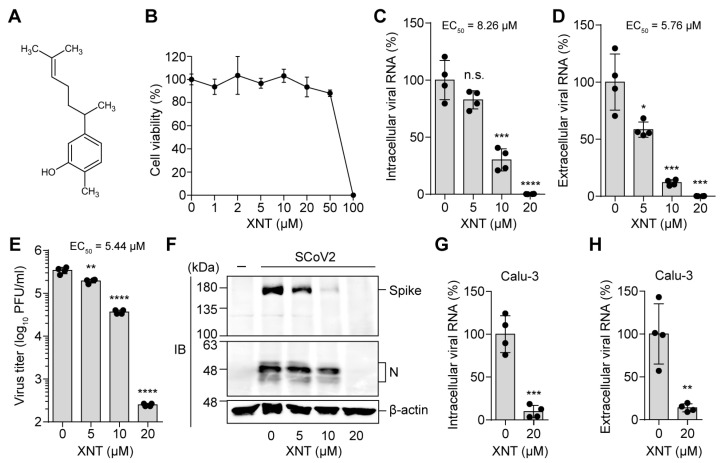
Antiviral activity of XNT against SCoV2. (**A**) Chemical structure of XNT. (**B**) Cytotoxicity of XNT, assessed by MTS assay 24 h after treatment of Vero E6 cells with increasing concentrations of XNT. The experiments were performed in biological triplicates. (**C**–**F**) Intracellular viral RNA (**C**), extracellular viral RNA (**D**), and infectious virus (**E**) titers, determined 24 h after treatment of SCoV2 (KCDC03)-infected Vero E6 cells (MOI = 0.01) with XNT. (**F**) shows the immunoblots for the indicated viral proteins in XNT-treated cells. (**G**,**H**) Antiviral activity of XNT in Calu-3 cells, assessed as described above. In (**C**–**H**), bar graphs show the mean ± SD with data points (two from each set) from two biological replicates. * *p* < 0.05; ** *p* < 0.01; *** *p* < 0.001; **** *p* < 0.0001; n.s., not significant; by unpaired two-tailed Student’s *t*-test.

We assessed the antiviral activity of XNT in SCoV2 (KCDC03)-infected, type I IFN-deficient Vero E6 cells [38], at a concentration range showing minimal cellular cytotoxicity (<20% cell viability reduction) (Figure 1A,B). XNT treatment led to a significant reduction of intracellular and extracellular viral RNA titers at 24 h post-infection (hpi), with an average mean EC_50_ of 8.26 μM and 5.76 μM, respectively (Figure 1C,D). Furthermore, XNT dose-dependently reduced infectious virus titer, with a ~3-log_10_ reduction at 20 μM (Figure 1E). Consequently, viral protein (Spike and N protein) expression was reduced substantially (Figure 1F). In SCoV2-permissive lung adenocarcinoma Calu-3 cells [39], similar degrees of reduction in intracellular and extracellular viral RNA copy numbers were achieved by 20 μM XNT (Figure 1G,H), without affecting cell viability (Supplementary Figure S1A). Further, extracellular viral RNA titer was decreased by > 2-log_10_-fold at 48 hpi of Calu-3 cells (Supplementary Figure S1B).

Of note, the antiviral potency of XNT was, however, >10-fold lower than that of the FDA-approved phosphoramidite nucleoside prodrug RDV, which was known to inhibit RNA synthesis by SCoV2 RNA polymerase [40], used as a positive control and for comparison of antiviral potency. Without affecting the cell viability at up to 20 μM (Supplementary Figure S2A), RDV dose-dependently inhibited viral replication, resulting in a significant reduction in intracellular viral RNA titers accompanied by a substantial reduction in viral protein expression (Supplementary Figure S2B,C). Its EC_50_ value of 0.33 μM was comparable to the antiviral efficacy (EC_50_ = 1.28 μM), determined by plaque assay [41].

### 3.2. Effect of XNT on SCoV2 Cellular Entry

**Figure 2:**
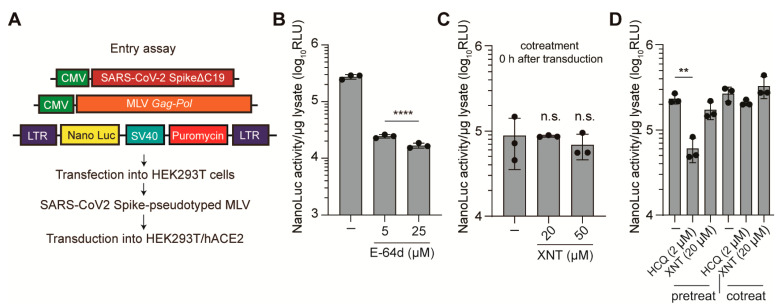
XNT does not interfere with SCoV2 entry. (**A**) Schematic diagrams of plasmids used for the generation of the SCoV2 spike protein-pseudotyped MLV. (**B**) Validation of the pseudotyped virus entry assay. Inhibition of the SCoV2 S protein-pseudotyped MLV by a cathepsin (a cysteine protease) inhibitor E-64d. (**C**,**D**) HEK293T cells transiently expressing hACE2 were treated with XNT or a lysosomotropic agent hydroxyclroquine (HCQ) known to block SCoV2 entry, 0 h after (co-treatment) or 1 h before (pre-treatment) transduction with the pseudotype virus, prior to determination of nano-luciferase activity at 60 h post-transduction. Data are mean ± SD from three independent experiments. (−), DMSO (0.1%) vehicle only-treated. ** *p* < 0.01; **** *p* < 0.0001; n.s., not significant.

To understand the mode of action of XNT, we first investigated whether it interferes with viral infectivity or entry by acting on virus particles or by perturbing cellular signaling pathways. We used a SCoV2 spike protein (S)-pseudotyped MLV that was generated by transfection of HEK293T cells with three different plasmids, which include an MLV packaging vector and plasmids individually expressing MLV *gag-pol* genes and SCoV2 S with a C-terminal 19 amino acids deletion (Figure 2A). Cellular entry of the resulting pseudotyped virus was inhibited by E-64d, a cathepsin inhibitor known to interfere with SCoV2 entry by blocking S-protein-mediated membrane fusion in the late endosome [42] (Figure 2B). Following validation of this surrogate model of SCoV2 entry, we tested if treatment of XNT during viral adsorption or its pretreatment (1 h before infection) inhibits cellular entry of the pseudotyped virus. The results showed that none of these treatments affected the cellular entry of the SCoV2 S-pseudotyped virus (Figure 2C). Notably, pretreatment, but not cotreatment, with HCQ (2 μM), a lysosomotropic agent previously shown to display antiviral activity against SCoV2 [42], reduced the luciferase activity (Figure 2D). By contrast, pretreatment with XNT (20 μM) had no effect on the viral entry. Altogether, these results suggest that the antiviral activity of XNT was not caused by interfering with the viral entry process.

### 3.3. Inhibition of SCoV1 Subgenomic Replicon Replication by XNT

**Figure 3:**
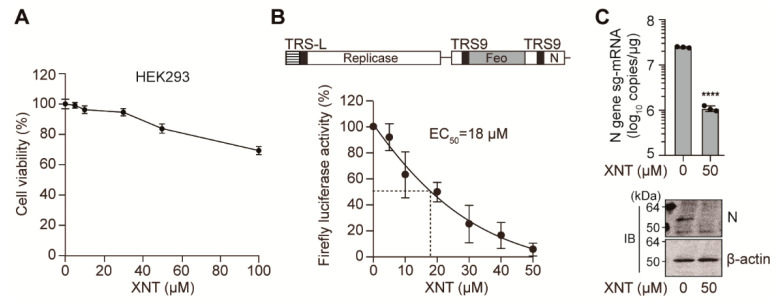
XNT inhibits SCoV1 replication. (**A**) Cytotoxicity of XNT in HEK293 cells, measured by MTS assay 24 h after treatment with increasing concentrations of XNT. (**B**,**C**) Inhibition of SCoV1 subgenomic replicon replication by XNT. HEK293 cells, co-transfected with pSARS-REP-Feo, a BAC-based SCoV1 subgenomic replicon-expressing plasmid along with pRL-TK used for transfection efficiency normalization. Six hours later, cells were treated with increasing concentrations of XNT for 24 h. Shown are the normalized luciferase activities relative to mock (0.1% DMSO vehicle)-treated cells (**B**). SCoV1 N-gene mRNA and N protein levels, determined by RT-qPCR and immunoblotting analysis (**C**). Feo, a Fluc gene fused to a neomycin phosphotransferase gene. TRS9, transcription-regulating sequence 9 required for synthesis of sg-mRNA9 of SCoV1. **** *p* < 0.0001; by unpaired two-tailed Student’s *t*-test.

Taking advantage of the fact that SCoV2 ORF1a/b, which produces 16 Nsps involved in the viral replication and evasion of the cellular antiviral defense system [5], is highly similar to SCoV1 in amino acid sequences of these Nsps, we tested if XNT can inhibit SCoV1 replication. In the range of XNT concentrations that reduced cell viability by less than 20% (Figure 3A), we evaluated its replication inhibitory activity using a SCoV1 subgenomic replicon that we established previously and used to evaluate the potency of viral replication inhibitory agents [23,43]. As shown in Figure 3B, XNT reduced Fluc activity, which is only expressed when the viral subgenomic mRNA encoding this reporter is produced following the replication of the replicon [23], by >90% at 50 μM, with an EC_50_ of 18 μM. The copy number of N gene-specific sg-mRNAs (N sg-mRNA) was also reduced by XNT treatment (Figure 3C). Consequently, N protein expression was reduced dramatically, demonstrating that the synthesis of these two subgenomic RNAs, which is directed by the transcription-regulating sequence 9 (TRS9) during replicon replication, was inhibited by XNT.

Notably, XNT treatment did not activate IFN-β expression in both HEK293 and Vero E6 cells (Supplementary Figure S3), suggesting that SCoV1 replicon inhibition was not caused by innate antiviral responses triggered by the type I IFN.

### 3.4. Inhibition of HCoV-229E by XNT

We asked whether XNT acts as a broad-spectrum antiviral agent that can be used for the treatment of a wide range of pathogenic RNA viruses. We addressed this possibility by testing its replication inhibitory activity using two well-established Huh7-derived cell lines harboring self-replicating viral subgenomic replicons of HCV and human norovirus (HuNoV). None of these replicons was inhibited by XNT (Supplementary Figure S4A,B). Furthermore, little antiviral activity was observed in murine norovirus-infected RAW264.7 cells; there was no reduction in both intracellular viral RNA and infectious virus titers at 24 hpi of this murine macrophage cells (Supplementary Figure S4C,D).

**Figure 4:**
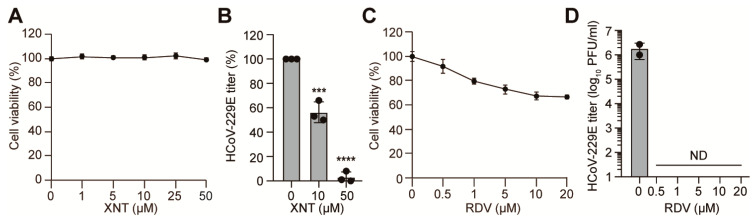
Comparison of antiviral activity of XNT and RDV against a common-cold-causing HCoV-229E. (**A**,**C**) Cytotoxicity of XNT (**A**) and RDV (**C**) in Huh7 cells, measured by MTS assay 48 h after treatment with increasing concentrations of the compounds. Data are mean ± SD from four biological repeats. (**B**,**D**) Infectious virus titers, determined 48 h after treatment of HCoV-229E-infected Huh7 cells (MOI = 0.0001) with XNT or RDV. ND, not detected. Bar graphs show the mean ± SD from biological replicates. In (**A**,**B**), the vehicle includes a final concentration of 0.1% DMSO. (**C**,**D**) Vehicle, 0.4% DMSO. RDV in each concentration has a final concentration of 0.4% DMSO. *** *p* < 0.001; **** *p* < 0.0001; by unpaired two-tailed Student’s *t*-test.

We then asked whether XNT shows antiviral activity against HCoV-229E, which is a less pathogenic HCoV responsible for the common cold, and is classified in the *Alphacoronavirus* genus, which evolutionally diverged from the *Betacoronavirus* genus to which SCoV1, MERS-CoV, and SCoV2 belong [5]. XNT did not reduce Huh7 cell viability at up to 50 μM (Figure 4A). Intriguingly, XNT treatment led to a dose-dependent reduction in viral loads in Huh7 cells infected with HCoV-229E at an MOI of 0.0001, with approximately 50% reduction in plaque formation at 10 μM (Figure 4B).

As observed with SCoV2, the antiviral efficacy of XNT against the HCoV was, however, lower than that of RDV. Notably, while no cytotoxicity was observed in Vero E6 cells (Supplementary Figure S2A), it reduced cell viability by >20% in Huh7 cells treated with >1 μM RDV (Figure 4C). Nevertheless, at a dose of 0.5 μM displaying less than 10% cell viability reduction, HCoV-229E was cleared to an undetectable level (Figure 4D). Altogether, our results underscore that XNT inhibits viral replication with a certain degree of selectivity toward SCoV and HCoV. Despite being less potent than the intravenous (iv) drug RDV, XNT may be used as an orally administrable herbal medicine inhibiting a wide range of CoVs.

### 3.5. Antiviral Efficacy of XNT against SCoV2 Variants

**Figure 5:**
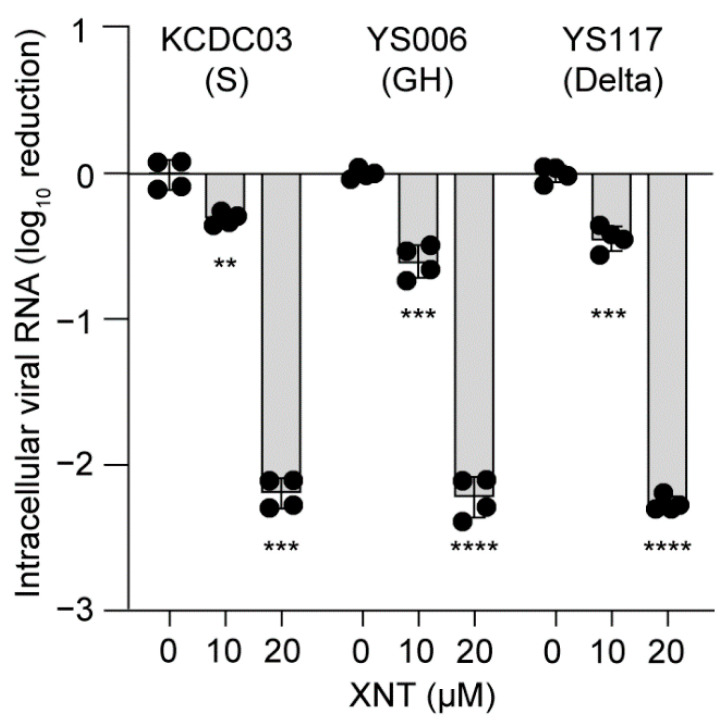
Inhibition of SCoV2 variants by XNT. Intracellular and extracellular viral RNA titers, determined 24 h after treatment of Vero E6 cells infected with SCoV2 KCDC03 (S clade), YS006 (GH clade), or a delta strain YS117 at an MOI of 0.01. Bar graphs show the mean with data points (two from each set) from two biological replicates. Vehicle, 0.1% DMSO. ** *p* < 0.01; *** *p* < 0.001; **** *p* < 0.0001; by unpaired two-tailed Student’s *t*-test.

Since the emergence of SCoV2 in late 2019, numerous variants have been isolated. Besides in the S protein, mutations have been also detected in their ORF1a/1b. The Wuhan/Hu-1/2019 or the strain we used in the present study KCDC03 (SARS-CoV-2/human/KOR/KCDC03/2020) differs from the GH clade strain YS006 (SARS-CoV-2/human/KOR/YS006/2020) in 5 different nsps (Nsp2, Nsp3, Nsp7, Nsp12, and Nsp16) encoded by the ORF1a/b [26] (Supplementary Table S2). Furthermore, between the delta strain YS117 (SARS-CoV-2/human/KOR/YS117/2021) and KCDC03 strain, more nonsynonymous mutations are present (13 amino acid changes in 6 different Nsps (Nsp3, Nsp4, Nsp6, Nsp12, Nsp13, and Nsp14); Supplementary Table S2). We reasoned that these variants might respond differently to XNT if these amino acid variations alter the binding of XNT to one of these nsps. The assessment of antiviral activity of XNT against these strains revealed that 20 μM XNT was capable of inhibiting SCoV2 variants equally well, with a >2-log_10_ reduction in intracellular viral RNA titer (Figure 5). These observations suggested a potential benefit of XNT against SCoV2 variants of concern, irrespective of the amino acid variations present in the variants we used.

### 3.6. XNT Lacks Inhibitory Activity against Nsp5, Nsp12, and Nsp13

CoVs encode three well-conserved viral enzymes, Nsp5, Nsp12, and Nsp13, which are essential for viral replication. The main protease (M^pro^ or 3CL protease) Nsp5 autocleaves itself and cuts the viral polyprotein on at least 11 cleavage sites to generate the Nsp12 RdRp and Nsp13 helicase [44]. Since XNT inhibited SCoV1 subgenomic replicon replication, we sought to identify its potential target(s) using in vitro enzyme assays for the Nsp12, Nsp5, and Nsp13, which show 96.4%, 96.1%, and 99.8% amino acid identity, respectively, between SCoV1 and SCoV2.

**Figure 6:**
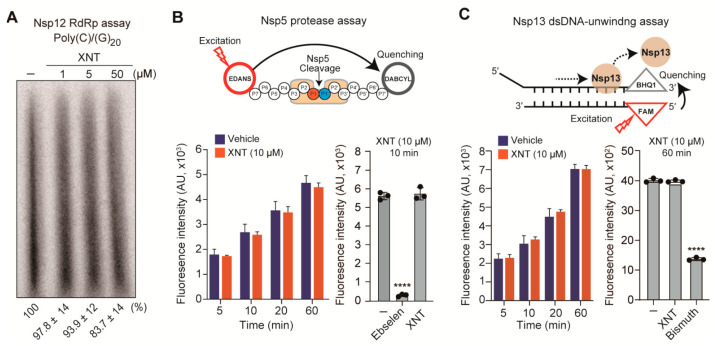
Analysis of inhibitory activity of XNT on SCoV2 enzymes. (**A**) SCoV2 Nsp12 (RdRp) inhibitory activity of XNT, evaluated by an in vitro RdRp assay with a poly(C) template in the presence of the oligo(rG)_20_ primer. Shown below are representative phosphorimager image from three independent experiments, which are signal intensities of radioisotope-labeled RNA products (mean ± SD from three independent experiments). (−), DMSO (4%) vehicle only-treated. (**B**) Schematic illustration of an in vitro FRET-based SCoV2 Nsp5 protease assay (top). Inhibitory activity of XNT and ebselen (10 μM each), determined by measuring fluorescence intensity of the cleaved peptide substrate bearing DABCYL (quencher) and EDANS (donor), for up to 60 min (bottom left) or for 10 min (bottom right). (**C**) Schematic illustration of the FRET-based SCoV2 Nsp13 helicase assay (top). Inhibitory activity of XNT and bismuth citrate (10 μM each), determined by measuring fluorescence signals emitted from the 5ʹ FAM of the single-strand DNA separated from the DNA duplex substrate with 5ʹ d(T)_20_ overhang and 3ʹ BHQ1 quencher, for up to 60 min (bottom left) or for 60 min (bottom right). Vehicle in (**B**,**C**), 1% DMSO (final concentration) in a reaction buffer. **** *p* < 0.0001; by unpaired two-tailed Student’s *t*-test.

As we previously reported [35], SCoV2 Nsp12 expressed in *E. coli* as a fusion protein with an N-terminal (His)_6_-tag was purified by affinity chromatography using a Ni-NTA column (Supplementary Figure S5A,B). The Nsp12, but not the one (Nsp12(SAA)) with an SAA substitution at the catalytic triad SDD within the RdRp active site, was found to have a primer-dependent RNA polymerizing activity on a homopolymeric RNA template poly(C) in the presence of a complementary primer (rG)_20_ (Supplementary Figure S5C), confirming our previous results obtained with SCoV1 Nsp12 [35]. The assay established with the functionally active SCoV2 Nsp12 revealed that XNT had little or no inhibitory effect on the Nsp12 RdRp activity (Figure 6A).

Using the FRET-based enzyme assays for the SCoV2 Nsp5 protease and Nsp13 RNA helicase (Supplementary Figures S6 and S7), we found that these two enzymes are not direct targets of XNT, while the protease activity of Nsp5 and the dsDNA-unwinding activity of Nsp13 were inhibited by ebselen and bismuth citrate, respectively (Figure 6B,C), as reported recently in other studies [45,46]. Taken together, these data suggest that the SCoV replication inhibitory activity of XNT is not caused by direct inhibition of Nsp5, Nsp12, and Nsp13, which are highly conserved in CoVs.

## 4. Discussion

The diverse biomedical activities of XNT along with molecular mechanisms behind its multiple bioactive properties have been explored [15]. However, to the best of our knowledge, there are no previous studies assessing the antiviral effects of XNT. In the present study, we show the antiviral activity of XNT against SCoV2. We also provide evidence that it displays selective antiviral activity against CoVs, including SCoV1 and HcoV-229E, with no significant effect on the replication of HCV and noroviruses (MNV and HuNoV).

While XNT inhibited ScoV1 subgenomic replicon replication, it is as yet unclear whether the antiviral efficacy was directed by inhibiting viral or cellular targets. We showed that at least Nsp12, Nsp5, and Nsp13, the three key enzymes required for CoV replication, were not significantly inhibited by XNT in in vitro assays. Besides these viral enzymes, multiple other Nsps, among a total of 16 Nsps encoded by the ORF1a/b of the CoVs, are also involved in viral RNA replication by forming a functional RNA replicase complex [47,48]. Particularly, Nsp7 and Nsp8, which are two accessory cofactors of Nsp12 [49], and the Nsp14 with a proofreading exonuclease activity might be a candidate Nsps targeted by XNT. Additional studies are required to understand how XNT inhibits CoV replication and identify its molecular targets. If XNT-resistant viral mutants can be selected, its target molecule and binding site might be predicted by a sequence analysis of the mutations conferring resistance to XNT and could be verified by reverse genetics studies using a SCoV2 infectious cDNA clone.

Apart from viral targets, XNT might target proviral host targets shared by the CoVs we tested in this study. Cyclin-dependent kinase-2 (CDK2) is a candidate target of XNT. CDK2 has been validated as a potential antiviral target for various viruses; CDK2-specific inhibitors or broad CDK family inhibitors showed inhibitory activity against HIV [50], herpes simplex virus, human adenovirus type-4, human cytomegalovirus, vaccinia virus, poxvirus [51], influenza A virus [52], and ZIKV [53] in culture systems, while detailed molecular mechanisms remain to be elucidated further. Interestingly, XNT treatment dose-dependently reduced CDK2 expression in the colon cancer cell HCT116 [54], suggesting CDK2 may be indirectly involved for XNT to display its broad antiviral activity. A recent study showed, by a phosphoproteomics analysis of SCoV2-infected cells, that CDK2 Thr14/Tyr15 phosphorylation increased at 2 hpi and decreased afterward [41]. It remained to be investigated if downregulation of CDK2 by XNT has any impact on the CDK2 phosphorylation profile and affects SCoV2 replication by preventing CDK2 activation, which appears to be triggered at the early stage of SCoV2 infection. Besides a well-known function in the regulation of the cell cycle, CDK2 was also shown to regulate NF-κB activation. Pharmacological inhibition of CDK2 depressed macrophage functions by reducing NF-κB activation [55]. It is worth noting that XNT inhibited interleukin-6 (IL-6) and tumor necrosis factor-α (TNF-α) production [56,57,58,59]. Considering that one of the severe symptoms of COVID-19 is cytokine storms caused by excess amounts of proinflammatory cytokines [60], this feature, among the multiple proposed modes of actions of XNT, may be an additional benefit in the management of COVID-19. More work is required to assess if this anti-inflammatory activity of XNT is, at least in part, mediated through the down-regulation of CDK2 by XNT, and can be beneficial irrespective of the stage of SCoV2 infection.

The activation of innate immune responses is another possible mechanism of the antiviral activity of XNT. Both SCoV2 and SCoV1 are known to be sensitive to type I IFNs, although the IFN-mediated canonical and noncanonical antiviral responses can be blunted by these CoVs [61]. A recent study predicted 20 potential molecular targets of XNT using a computational target fishing approach [62]. Some of the predicted host targets of XNT appear to be linked to antiviral responses triggered by type I IFN. For instance, histone deacetylase 8 (HDAC8) inhibition by XNT might explain its broad-spectrum antiviral activity as HDAC8 was previously proposed as a repressor of IFN-β [63]. In fact, HDAC8 inhibitors showed antiviral effects against herpes simplex virus-1 [64] and influenza A virus [65]. However, this hypothesis is contradictory to our findings of the lack of inhibitory activity of XNT against noroviruses, which are sensitive to type I IFNs [29]. Furthermore, because XNT displays antiviral activity in Vero E6 cells, which are defective in type I IFN production [38], its mode of action should be independent of type I IFN production. Additionally, our results showed that XNT per se is not an inducer of IFN-β production (Supplementary Figure S3). These results rule out the possibility that XNT activates the IFN-inducing or IFN-signaling pathway to exert its antiviral activity.

*C. xanthorrhiza* has been traditionally used not only as herbal medicine, but also been consumed widely as tea and food for centuries. No signs of toxicity were observed in mice following the oral administration of 2000 mg/kg body weight of *C. xanthorrhiza* extract [66] or a single compound XNT at a concentration of 500 mg/kg [67]. ADME (absorption, distribution, metabolism, excretion)-related properties of XNT support the drug-likeness of XNT [62]. With its safety profile in pre-clinical trials in mice [15,67,68], clinical testing of its antiviral efficacy against SCoV2 and HCoV is warranted.

## 5. Conclusions

In summary, XNT is an attractive antiviral candidate for treating SARS-CoV-2 and related CoVs. The mechanism of the observed antiviral activity in a broad range of CoVs in the genus of *Alphacoronavirus* and *Betacoronavirus* could not be elucidated in the present study, but is likely to be associated with viral RNA replication. It is possible that inhibition of nsps of CoVs, other than the ones (Nsp5, Nsp12, and Nsp13) we tested in this study, might account for the selective inhibition of CoVs by XNT. Considering its availability in large quantities, relatively easy ways for mass production through chemical synthesis or agricultural farming, and affordability, XNT might find its merit of further investigation as an orally administrable herbal medicine with potential pan-CoV antiviral activity.

## Data Availability

The data presented in this manuscript and all other relevant data supporting the findings of this study are available from the corresponding author upon reasonable request.

## Supplementary Materials

The following are available on at https://www.mdpi.com/article/10.3390/biomedicines9111725/s1, Figure S1: SCoV2 inhibitory activity of XNT in Calu-3 cells. Figure S2: Antiviral activity of remdesivir (RDV) against SCoV2. Figure S3: XNT treatment does not induce interferon β production. Figure S4: Selective inhibition of SCoV replication by XNT. Figure S5: Expression and purification of a full-length, functionally active SCoV2 Nsp12 protein. Figure S6: An in vitro SCoV2 Nsp5 protease assay using a purified Nsp5 protein. Figure S7: An in vitro SCoV2 Nsp13 helicase assay using a purified Nsp13 protein. Table S1: Primer sets used for quantification of viral RNA levels by RT-qPCR. Table S2: Differences in ORF1ab amino acids between three SCoV2 strains used in this study.
