# Supplementary material for: In Vitro Replication Inhibitory Activity of Xanthorrhizol against Severe Acute Respiratory Syndrome Coronavirus 2
Source: Biomedicines. 2021 Nov 19;9(11):1725. doi: 10.3390/biomedicines9111725 (PMC8615586; doi:10.3390/biomedicines9111725)
Supplement: Supplementary file 1 [file biomedicines-09-01725-s001.zip › biomedicines-1457279-supplementary.pdf]

Supplementary Materials

In Vitro Replication Inhibitory Activity of Xanthorrhizol against Severe Acute Respiratory Syndrome Coronavirus 2

Minwoo Kim [1,†], Hee Cho [1,†], Dae-Gyun Ahn [1,‡], Hae-Gwang Jung [1], Han Young Seo [1], Ji-Su Kim [1], Youn-Jung Lee [2], Jun Yong Choi [2], In Ho Park [3], Jeon-Soo Shin [3,4], Seong-Jun Kim [5] and Jong-Won Oh [1,*]

[1]*Department of Biotechnology, Yonsei University, 50 Yonsei-ro, Seodaemun-gu, Seoul 03722, Korea*
[2]*Division of Infectious Diseases, Department of Internal Medicine, Yonsei University College of Medicine, Seoul 03722, Korea*
[3]*Severance Biomedical Science Institute, Yonsei University College of Medicine, Seoul 03722, Korea*
[4]*Department of Microbiology, Yonsei University College of Medicine, Seoul 03722, Korea*
[5]*Center for Convergent Research of Emerging Virus Infection, Korea Research Institute of Chemical Technology, Daejeon 34114, Korea*

[†]These authors contributed equally to this work.
[‡]Current address: Center for Convergent Research of Emerging Virus Infection, Korea Research Institute of Chemical Technology, Daejeon 34114, Korea.

Contents:
Supplementary Tables 1 and 2
Supplementary Figures 1–7
Supplementary References

*Correspondence should be addressed to J.-W.O. (jwoh@yonsei.ac.kr).

Supplementary Tables

Supplementary Table S1. Primer sets used for quantification of viral RNA levels by RT-qPCR

Gene	Primer set	Sequence (5′–3′)[a]	Ref.
SARS-CoV-2	Forward	CCCTGTGGGTTTTACACTTAA	[1]
	Reverse	ACGATTGTGCATCAGCTGA	
	Probe	FAM-CCGTCTGCGGTATGTGGAAAGGTTATGG-BHQ1	
SARS-CoV N	Forward	ATATTAGGTTTTTACCTACCCAGG	[2]
	Reverse	CGTCGGGTAGCTCTTCGGTAG	
	Probe	FAM-TCCTCCTTGCCATGCTGAGTGAGA-BHQ1	
HCV replicon	Forward	GCGTCTAGCCATGGCCTTAGTATGAGTGTC	[3]
	Reverse	ACCACAAGGCCTTTCGCGACCCAACACTAC	
	Probe	FAM-CTGCGGAACCGGTGAGTACAC-TAMRA	
MNV-1	Forward	CACGCCACCGATCTGTTCTG	[4]
	Reverse	GCGCTGCGCCATCACTC	
NoV-GI	Forward	CGYTGGATGCGNTTYCATGA[a]	[5]
	Reverse	CTTAGACGCCATCATCATTYAC[a]	
hGAPDH	Forward	GAAGGTGAAGGTCGGAGTC	[6]
	Reverse	GAAGATGGTGATGGGATTTC	

[a] Mixed bases are as follows: Y, C or T; N, any. NoV-GI, human norovirus genotype I.

Supplementary Table S2. Differences in ORF1ab amino acids between three SCoV2 strains used in this study

Gene name	a.a. position	Wuhan/Hu-1[a]	KCDC03 (S)	YS006 (GH)	YS117 (Delta)
nsp2	85	Thr	Thr	**Ile** [b]	Thr
nsp3	77	Leu	Phe	**Leu**	**Leu**
	707	Ile	Ile	Ile	**Leu**
	792	His	His	His	**Leu**
	822	Pro	Pro	Pro	**Leu**
nsp4	293	Val	Val	Val	**Ile**
	446	Ala	Ala	Ala	**Val**
nsp6	149	Val	Val	Val	**Ala**
	181	Thr	Thr	Thr	**Ile**
nsp7	25	Ser	Ser	**Leu**	Ser
nsp12	323	Pro	Pro	**Leu**	**Leu**
	671	Gly	Gly	Gly	**Ser**
nsp13	77	Pro	Pro	Pro	**Leu**
	210	Val	Val	Val	**Ile**
nsp14	394	Ala	Ala	Ala	**Val**
nsp16	6	Gln	Gln	**Leu**	Gln

[a] Wuhan/Hu-1, MN988668; KCDC03, MT020782; YS006, MW345824; YS117, MZ798798

[b] Amino acid changes in the YS006 and YS117, in comparison to the reference strain KCDC003, are in bold-type.

Supplementary Figures

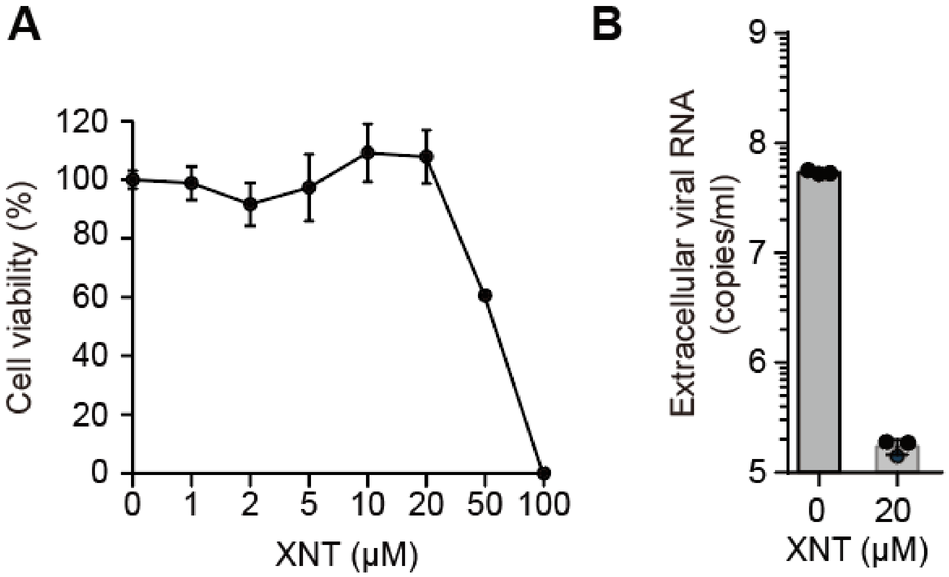

Supplementary Figure S1. SCoV2 inhibitory activity of XNT in Calu-3 cells. (**A**) Cytotoxicity of XNT, assessed by MTS assay 24 h after treatment of Calu-3 cells with increasing concentrations of XNT. The experiments were performed in biological triplicates. (**B**) Extracellular viral RNA titers, determined 48 h after treatment of SCoV2-infected Calu-3 cells (MOI = 0.01) with 20 µM XNT. Bars represent mean values ± SD, and dots represent three biological replicates.

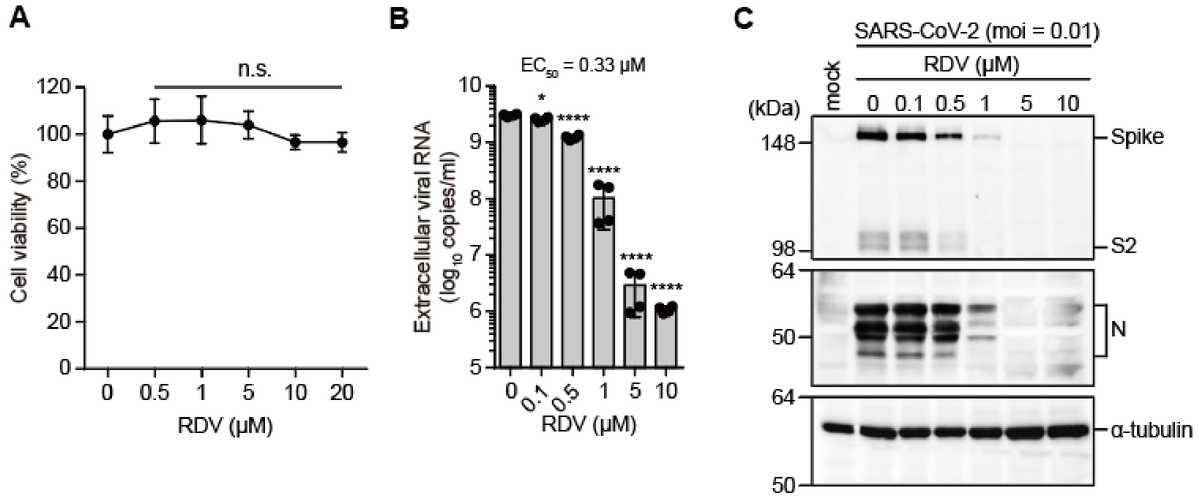

Supplementary Figure S2. Antiviral activity of remdesivir (RDV) against SCoV2. (**A**) Cytotoxicity of RDV, assessed by MTS assay 48 h after treatment of Vero E6 cells with increasing concentrations of RDV. Data are mean ± SD from biological triplicates. (**B** and **C**) Extracellular viral RNA titers, determined 48 h after treatment of SARS-CoV-2-infected Vero E6 cells (MOI = 0.01) with increasing concentrations of RDV (**B**). Immunoblots for the indicated viral proteins are shown below bar graph (**C**). In (**B**), bars represent mean values, and dots represent two biological replicates conducted with technical duplicates. $*p < 0.05$; $****P < 0.0001$; n.s., not significant; by unpaired two-tailed Student's *t*-test

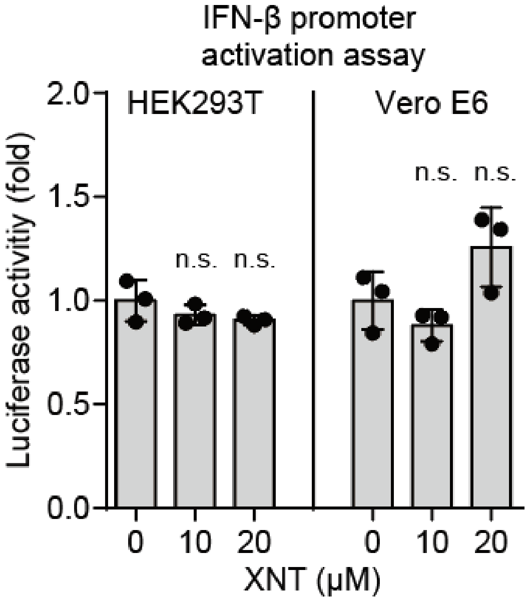

Supplementary Figure S3. XNT treatment does not induce interferon β production. Firefly luciferase (Fluc) expression under the control of *IFN-β* promoter was determined 24 h after treatment with XNT following transfection of HEK293T or Vero E6 cells with the assay reporter. Shown are the fold-changes of normalized Fluc activity relative to DMSO (0.1%) vehicle-treated control. Bars represent mean values from biological triplicates, and each dot represent the average of two technical replicates (each from biological triplicates). n.s., not significant; by unpaired two-tailed Student's *t*-test.

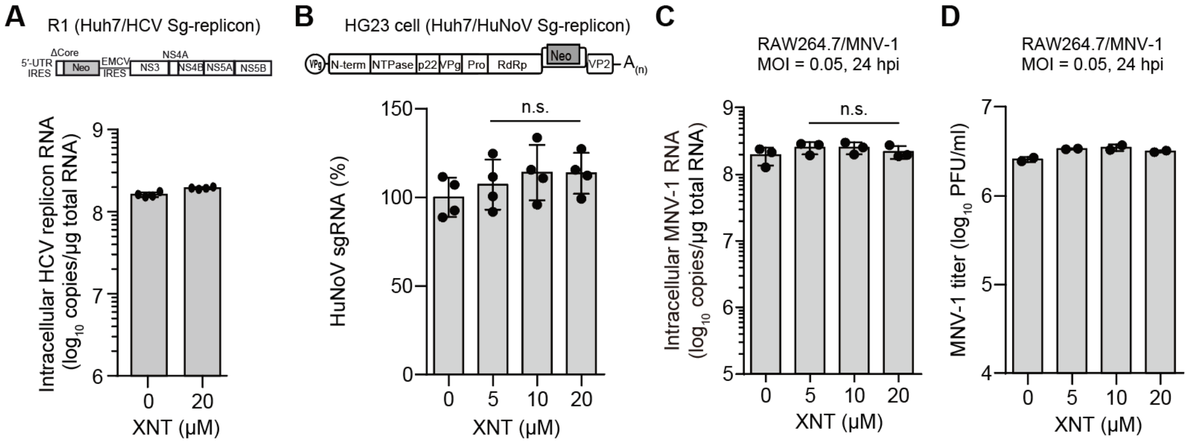

Supplementary Figure S4. Selective inhibition of SCoV replication by XNT. (**A** and **B**) Antiviral activity of XNT in R-1 and HG23 cell lines, which harbor a self-replicating HCV (**A**) and human norovirus (**B**) subgenomic replicon, respectively, assessed by RT-qPCR quantification of intracellular replicon RNA titer 2 days after XNT treatment. Data in (**A**) are shown as mean ± SD from biological quadruplicates, each with two technical replicates. Each dot represents the average of two technical replicates. In (**B**), dots represent data points from two biological replicates, each with two technical replicates. (**C** and **D**) Intracellular MNV-1 genome copy number (**C**) and virus loads (**D**), determined 24 h after infection of RAW264.7 cells with MNV-1 (MOI = 0.05). In (**C**), bars represent mean values, and dots represent the averages of two technical replicates, each from biological triplicates. In (**D**), bars represent averages values, and dots represent two technical replicates for a representative experiment. n.s., not significant; by unpaired two-tailed Student's *t*-test.

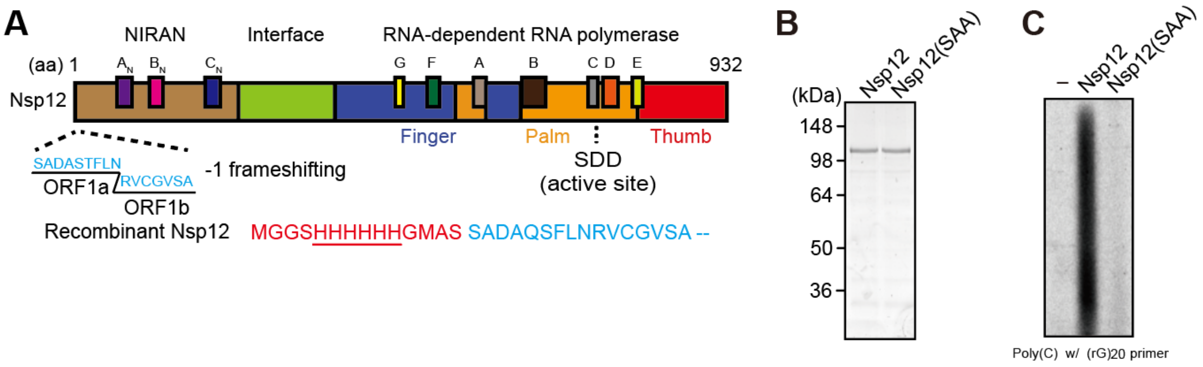

Supplementary Figure S5. Expression and purification of a full-length, functionally active SCoV2 Nsp12 protein. (**A**) Schematic of the recombinant N-terminal $(His)_6$-tagged Nsp12 protein. The authentic Nsp12 amino acids sequence and extra-amino acids added during cloning procedure are shown in blue and red, respectively. (**B**) Purified wild-type Nsp12 and its inactive mutant Nsp12(SAA) resolved by SDS-PAGE and stained with Coomassie blue. (**C**) Autoradiography of radioisotope-labeled RNA products produced from a poly(C) RNA template in the presence of the $(rG)_{20}$ primer. Quantification data are means ± SD from three independent experiments.

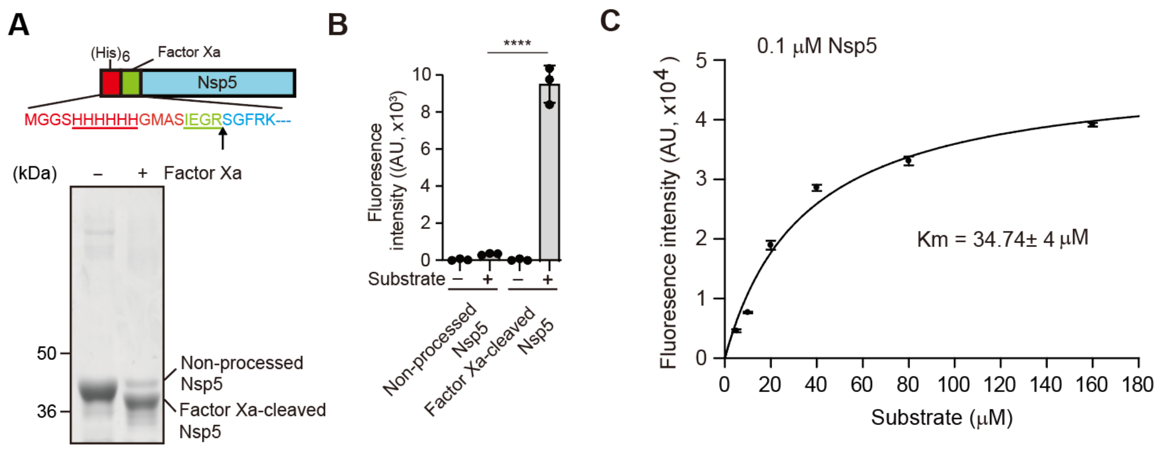

Supplementary Figure S6. An *in vitro* SCoV2 Nsp5 protease assay using a purified Nsp5 protein. (**A**) Schematic of the N-terminal $(His)_6$-tagged recombinant Nsp5 protein. The authentic Nsp5 amino acids sequence and extra-amino acids added during cloning procedure are shown in blue and red, respectively, with the factor Xa-cleavage site indicated by an arrow. Shown below is the factor Xa-processed recombinant protein, visualized by staining with Coomassie blue following SDS-PAGE. (**B**) A FRET-based Nsp5 protease assay demonstrating that removal of the N-terminal fusion part by factor-Xa treatment is critical to confer enzyme activity to the processed form. Data are means ± SD from three independent experiments. ****$P < 0.0001$; by unpaired two-tailed Student's *t*-test. (**C**) Determination of Km of the purified Nsp5 protein.

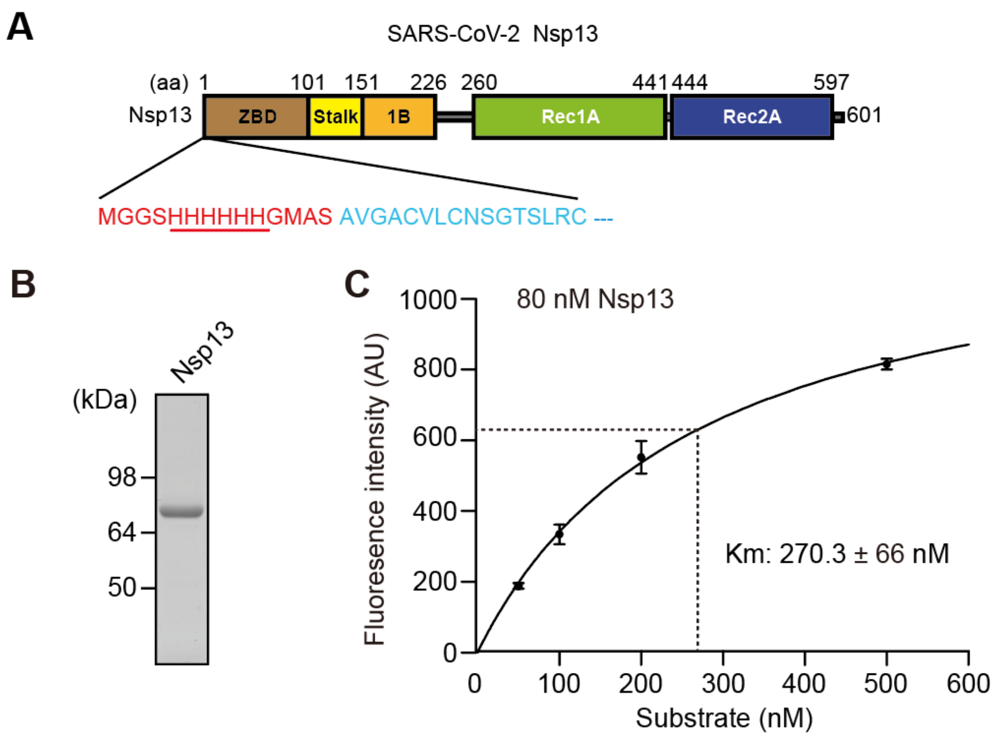

Supplementary Figure S7. An *in vitro* SCoV2 Nsp13 helicase assay using a purified Nsp13 protein. (**A**) Schematic of the recombinant N-terminal $(His)_6$-tagged Nsp13 protein. The authentic Nsp13 amino acids sequence and extra-amino acids added during cloning procedure are shown in blue and red, respectively. Shown below is the purified Nsp13 recombinant protein used in the assay. (**B**) Determination of Km of SCoV2 Nsp13 protein. Data are means ± SD from three independent experiments.

Supplementary References

1. National Institute for Viral Disease Control and Prevention (2020) Specific primers and probes for detection 2019 novel coronavirus, http://ivdc.chinacdc.cn/kyjz/202001/t20200121_211337.html (accessed on 10 October 2021).
2. Ahn, D.G.; Lee, W.; Choi, J.K.; Kim, S.J.; Plant, E.P.; Almazan, F.; Taylor, D.R.; Enjuanes, L.; Oh, J.W. Interference of ribosomal frameshifting by antisense peptide nucleic acids suppresses SARS coronavirus replication. *Antiviral Res.* **2011**, *91*, 1-10.
3. Kim, S.J.; Kim, J.H.; Kim, Y.G.; Lim, H.S.; Oh, J.W. Protein kinase C-related kinase 2 regulates hepatitis C virus RNA polymerase function by phosphorylation. *J. Biol. Chem.* **2004**, *279*, 50031-50041.
4. Baert, L.; Wobus Christiane, E.; Van Coillie, E.; Thackray Larissa, B.; Debevere, J.; Uyttendaele, M. Detection of murine norovirus 1 by using plaque assay, transfection assay, and real-time reverse transcription-PCR before and after heat exposure. *Appl. Environ. Microbiol.* **2008**, *74*, 543-546.
5. Kageyama, T.; Kojima, S.; Shinohara, M.; Uchida, K.; Fukushi, S.; Hoshino, F.B.; Takeda, N.; Katayama, K. Broadly reactive and highly sensitive assay for Norwalk-like viruses based on real-time quantitative reverse transcription-PCR. *J. Clin. Microbiol.* **2003**, *41*, 1548-1557.
6. Lee, W.; Kim, M.; Lee, S.H.; Jung, H.G.; Oh, J.W. Prophylactic efficacy of orally administered *Bacillus* poly-γ-glutamic acid, a non-LPS TLR4 ligand, against norovirus infection in mice. *Sci. Rep.* **2018**, *8*, 8667.
